# Feline herpesvirus type 1 infection alters the diversity of upper respiratory tract microbiota in cats

**DOI:** 10.3389/fvets.2025.1663056

**Published:** 2025-08-22

**Authors:** Liyang Li, Wen Feng, Lanxinyue Bi, Ganzhen Deng

**Affiliations:** Department of Clinical Veterinary Medicine, College of Veterinary Medicine, Huazhong Agricultural University, Wuhan, China

**Keywords:** feline herpesvirus type 1, upper respiratory tract, microbiota, diversity, 16S rRNA sequencing

## Abstract

**Introduction:**

Feline herpesvirus type 1 (FHV-1) is a primary pathogen causing feline upper respiratory tract diseases (FURTD), but its impact on the upper respiratory tract microbiota remains unclear. This study aimed to evaluate the impact of FHV-1 infection on the upper respiratory tract microbiota by comparing the microbial composition between FHV-1-positive group with FHV-1-negative group.

**Methods:**

The microbial diversity in the upper respiratory tract of FHV-1-positive cats (*n* = 8) were analyzed using 16S rRNA high-throughput sequencing, and then this diversity was compared with that in healthy FHV-1-negative controls (*n* = 4).

**Results:**

Sequencing results showed that FHV-1 infection significantly increased microbial diversity (Shannon index: 5.55 ± 0.17 vs. 5.30 ± 0.11, *p* < 0.05; Simpson index: 0.95 ± 0.01 vs. 0.94 ± 0.00, *p* < 0.01) and altered community structure, as indicated by beta diversity analysis. At the phylum level, *Actinobacteriota* showed significantly higher relative abundance in the FHV-1-positive group than in the FHV-1-negative control group (*p* < 0.05). For the genus level, *Porphyromonas* and *Bergeyella* were significantly less abundant in FHV-1-positive group versus FHV-1-negative healthy control group (*p* < 0.05). Linear discriminant analysis effect size (LEfSe) identified *Prevotellaceae* as a biomarker for the FHV-1-positive group.

**Discussion:**

This study provided the first evidence that FHV-1 infection significantly alters the diversity and composition of the upper respiratory tract microbiota in cats. These microbiota changes were likely to play an important role in the pathogenesis of FURTD and offer new targets for the development of microbiota-based therapeutic strategies.

## 1 Introduction

Feline herpesvirus type 1 (FHV-1) is one of the most prevalent etiological agents of feline upper respiratory tract disease (FURTD), and has a global endemic presence (1). Epidemiological investigations demonstrate that the global prevalence of FHV-1 infection ranges from 20.9 to 55.26% in domestic cat populations, with significantly higher infection rates observed in high-density environments such as multi-cat households and animal shelters (2–4). Infected cats typically manifest classic symptoms, including conjunctivitis, rhinitis, and tracheitis (5). Severe cases may progress to corneal ulcers, pneumonia, or even death, posing a substantial threat to the global cat breeding industry and feline pet health (6, 7). Existing studies have demonstrated that cats infected with FHV-1 will maintain lifelong viral persistence (2). Following primary infection, FHV-1 typically establishes latency in the trigeminal ganglia and mandibular nerves of affected cats without causing clinical manifestations. However, viral reactivation and subsequent replication may occur during periods of immunosuppression or environmental stress, leading to recurrent clinical disease (8).

The upper respiratory microbiota, serving as the first line of defense against pathogens, engages in intricate symbiotic interactions with the host (9). Research in human medicine has demonstrated that respiratory viral infections can significantly disrupt microbial diversity, promote the overgrowth of opportunistic pathogens, and exacerbate tissue damage and systemic inflammation (10). Similarly, studies on feline calicivirus (FCV) infection have linked oral dysbiosis to elevated levels of pro-inflammatory cytokines, including IL-1β and TNF-α (11). However, whether FHV-1 infection similarly disrupts the upper respiratory microbiota and contributes to disease progression remains unclear. Despite advancements in understanding FHV-1 infection mechanisms and its interaction with host immunity, the dynamic changes in the upper respiratory microbiota following FHV-1 infection, key microbial taxa, and the mechanisms of microbe-immune interactions remain undefined (12).

This study aimed to compare the microbial diversity between FHV-1-positive and healthy cats, identify differentially abundant taxa and potential biomarkers, and explore the clinical implications of these findings for FURTD management. By addressing these objectives, our research endeavors to provide novel theoretical foundations and innovative strategies for clinical diagnosis and treatment.

## 2 Materials and methods

### 2.1 Sample collection and grouping

All samples for this study were derived from an epidemiological survey of feline upper respiratory tract disease conducted in Wuhan between 2021 and 2024, encompassing a total of 1,427 cases (Supplementary material 1). From this dataset, we selected 8 FHV-1-positive samples based on stringent inclusion criteria, ensuring all specimens were obtained from adult British Shorthair cats exhibiting upper respiratory symptoms, housed in single-cat household, and having received routine vaccinations. The cohort maintained a balanced gender distribution (4 males and 4 females), with laboratory confirmation via RT-qPCR confirming FHV-1 positivity while excluding co-infections with feline calicivirus (FCV), *Mycoplasma felis (Mf)*, *Chlamydia felis (Cf)*, and *Bordetella bronchiseptica (Bb)*. Additionally, four FHV-1-negative control samples were included, sourced from asymptomatic adult British Shorthairs under identical conditions (single-cat households, vaccinated, 2 males and 2 females), with RT-qPCR verification confirming the absence of FHV-1, FCV, *Mf*, *Cf*, and *Bb*. All samples were collected from animals that had not received antimicrobial treatment within the preceding week (Table 1).

**Table 1:** Basic information of the samples.

Sample ID	Gender	Breed	Age	Vaccination status	Household structure	FHV-1	FCV	*M f*	*Cf*	*Bb*
FHV 1	Female	British Shorthair	Adult	Vaccinated	Single cat household	Positive	Negative	Negative	Negative	Negative
FHV 2	Male	British Shorthair	Adult	Vaccinated	Single cat household	Positive	Negative	Negative	Negative	Negative
FHV 3	Female	British Shorthair	Adult	Vaccinated	Single cat household	Positive	Negative	Negative	Negative	Negative
FHV 4	Female	British Shorthair	Adult	Vaccinated	Single cat household	Positive	Negative	Negative	Negative	Negative
FHV 5	Male	British Shorthair	Adult	Vaccinated	Single cat household	Positive	Negative	Negative	Negative	Negative
FHV 6	Male	British Shorthair	Adult	Vaccinated	Single cat household	Positive	Negative	Negative	Negative	Negative
FHV 7	Male	British Shorthair	Adult	Vaccinated	Single cat household	Positive	Negative	Negative	Negative	Negative
FHV 8	Female	British Shorthair	Adult	Vaccinated	Single cat household	Positive	Negative	Negative	Negative	Negative
Healthy 1	Female	British Shorthair	Adult	Vaccinated	Single cat household	Negative	Negative	Negative	Negative	Negative
Healthy 2	Female	British Shorthair	Adult	Vaccinated	Single cat household	Negative	Negative	Negative	Negative	Negative
Healthy 3	Male	British Shorthair	Adult	Vaccinated	Single cat household	Negative	Negative	Negative	Negative	Negative
Healthy 4	Male	British Shorthair	Adult	Vaccinated	Single cat household	Negative	Negative	Negative	Negative	Negative

The sterile swabs were inserted transnasally to the nasopharynx (depth adjusted by weight: 4–5 cm for <3 kg, 5–7 cm for ≥3 kg), rotated three times against the posterior pharyngeal wall, and immediately preserved in RNA/DNA stabilization solution at −80°C. All procedures complied with the Guide for the Care and Use of Laboratory Animals (National Research Council, 2011) and were approved by the Institutional Animal Care and Use Committee of HZAU (protocol code: HZAUCA-2025-0038). Owners provided written consent for sample collection and data use, including potential risks and benefits.

### 2.2 High-throughput 16S rRNA gene sequencing

Total DNA was extracted using the TGuide S96 Magnetic Soil/Feces DNA Kit (Tiangen Biotech, China) with negative controls to exclude contamination (sterile ultrapure water) according to manufacturer’s instructions. The quality and quantity of the extracted DNA were examined using electrophoresis on a 1.8% agarose gel, and DNA concentration and purity were determined with NanoDrop 2000 UV–Vis spectrophotometer (Thermo Scientific, USA). The full-length 16S rRNA gene were amplified with primer pairs 27F: AGRGTTTGATYNTGGCTCAG and 1492R: TASGGHTACCTTGTTASGACTT. Both the forward and reverse 16S primers were tailed with sample-specific PacBio barcode sequences to allow for multiplexed sequencing. We chose to use barcoded primers because this reduces chimera formation as compared to the alternative protocol in which primers are added in a second PCR reaction. The KOD One PCR Master Mix (TOYOBOLife Science, Japan) was used to perform 25 cycles of PCR amplification, with initial denaturation at 95°C for 2 min, followed by 25 cycles of denaturation at 98°C for 10 s, annealing at 55°C for 30 s, and extension at 72°C for 1 min 30 s, and a final step at 72°C for 2 min. The total of PCR amplicons was purified with VAHTSTM DNA Clean Beads (Vazyme, China) and quantified using the Qubit dsDNA HS Assay Kit and Qubit 3.0 Fluorometer (Thermo Fisher Scientific, USA). After the individual quantification step, amplicons were pooled in equal amounts. SMRTbell libraries were prepared from the amplified DNA by SMRTbell Express Template Prep Kit 2.0 according to the manufacturer’s instructions (Pacific Biosciences, USA). Purified SMRTbell libraries from the pooled and barcoded samples were sequenced on a PacBio Sequel II platform (Beijing Biomarker Technologies, China) using Sequel II binding kit 2.0.

### 2.3 Bioinformatics analysis

The bioinformatics analysis of this study was performed with the aid of the BMKCloud.^1^ The raw reads generated from sequencing were filtered and demultiplexed using the SMRT Link software (version 8.0) with the minPasses ≥5 and minPredicted Accuracy ≥0.9, in order to obtain the circular consensus sequencing (CCS) reads. Subsequently, the lima (version 1.7.0) was employed to assign the CCS sequences to the corresponding samples based on their barcodes. CCS reads containing no primers and those reads beyond the length range (1,200–1,650 bp) were discarded through the recognition of forward and reverse primers and quality filtering using the Cutadapt (version 2.7) quality control process. The UCHIME algorithm (v8.1) was used in detecting and removing chimera sequences to obtain the clean reads. Sequences with similarity > 97% were clustered into the same operational taxonomic unit (OTU) by USEARCH (v10.0), and the OTUs < 2 in all samples were filtered.

Clean reads then were conducted on feature classification to output ASVs (amplicon sequence variants) by DADA2, and the ASVs < 2 in all samples were filtered. Taxonomy annotation of the OTUs/ASVs was performed based on the Naive Bayes classifier in QIIME2 using the SILVA database (release 138.1) with a confidence threshold of 70%. The Alpha diversity was calculated and displayed by the QIIME2 and R software, respectively. Beta diversity was determined to evaluate the degree of similarity of microbial communities from different samples using QIIME. The Permutational Multivariate Analysis of Variance (PERMANOVA) analyses were performed using the vegan package in R, with visualizations generated in Python. Prior to the above analysis, rarefaction curves and rank abundance curves were generated to assess the sequencing depth. Furthermore, differential taxa identification between groups was performed using Linear discriminant analysis effect size (LEfSe) analysis with a Linear Discriminant Analysis (LDA) score threshold of 2.0 and a *p*-value cutoff of < 0.05, combined with Wilcoxon rank-sum tests to validate group-specific differences in individual taxonomic units. For dominant genera with relatively high abundance (relative abundance > 1%), further validation was conducted using ANOVA tests for normally distributed data or Kruskal-Wallis tests for non-normally distributed data. *p*-values were corrected using the Benjamini-Hochberg method to control the false discovery rate.

## 3 Results

### 3.1 Analysis of sequencing sequence and OTUs number

**Figure 1:**
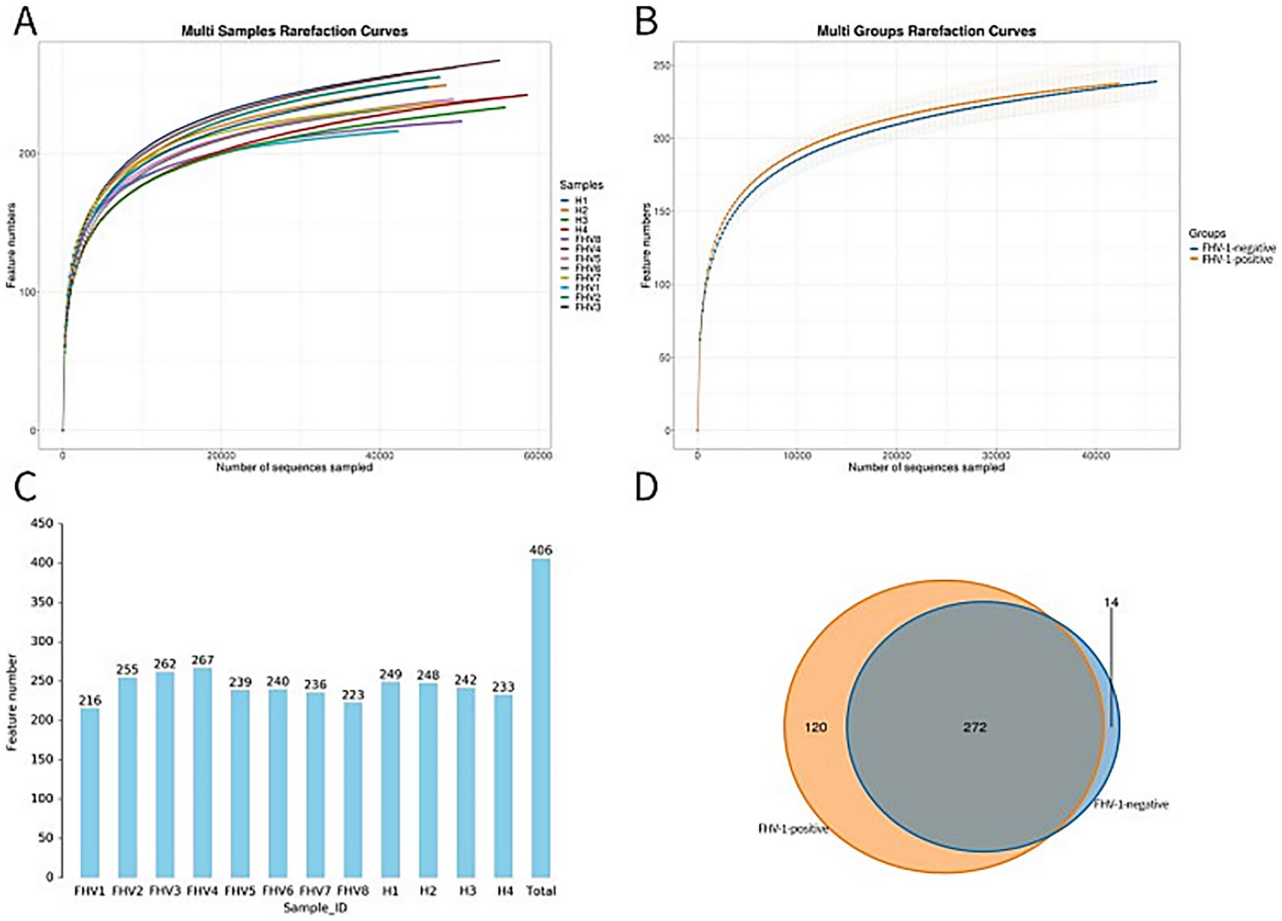
Display of OTU clustering results. **(A)** Multi samples rarefaction curves. **(B)** Multi groups rarefaction curves. **(C)** Number of OTUs in each sample. **(D)** Venn diagram of OTU distribution between FHV-1-positive and FHV-1-negative cats.

Sequencing results showed that the FHV-1-positive group obtained an average of 51,679.375 effective CCS, while the FHV-1-negative group obtained an average of 54,363.25 effective CCS (Table 2). The rarefaction curve results indicated that all curves approached a saturation trend, suggesting that the number of species in this environment would not increase significantly with the increase in sequencing depth (Figures 1A,B). At 97% sequence similarity threshold, microbial community analysis identified 272 shared OTUs between FHV-1-positive group and FHV-1-negative group. FHV-1-positive group harbored 120 unique OTUs, whereas FHV-1-negative group contained 14 unique OTUs (Figures 1C,D). These findings demonstrate significant disparities in microbial diversity between the two groups, with FHV-1-positive group exhibiting remarkably higher microbial diversity than FHV-1-negative group.

**Table 2:** Statistics of sample sequencing data processing results.

Sample ID	Raw CCS	Clean CCS	Effective CCS	AvgLen(bp)	Effective
FHV 1	46,991	46,942	43,591	1,453	92.76
FHV 2	56,313	56,239	49,123	1,449	87.23
FHV 3	60,617	60,485	52,958	1,450	87.36
FHV 4	64,642	64,510	58,138	1,447	89.94
FHV 5	55,936	55,849	51,680	1,451	92.39
FHV 6	60,449	60,390	56,197	1,453	92.97
FHV 7	56,547	56,515	49,984	1,451	88.39
FHV 8	57,207	57,172	51,764	1,452	90.49
Healthy 1	57,373	57,248	51,408	1,452	89.6
Healthy 2	55,503	55,400	49,111	1,451	88.48
Healthy 3	65,959	65,862	59,974	1,453	90.93
Healthy 4	62,550	62,464	56,960	1,453	91.06

### 3.2 Alpha diversity

**Figure 2:**
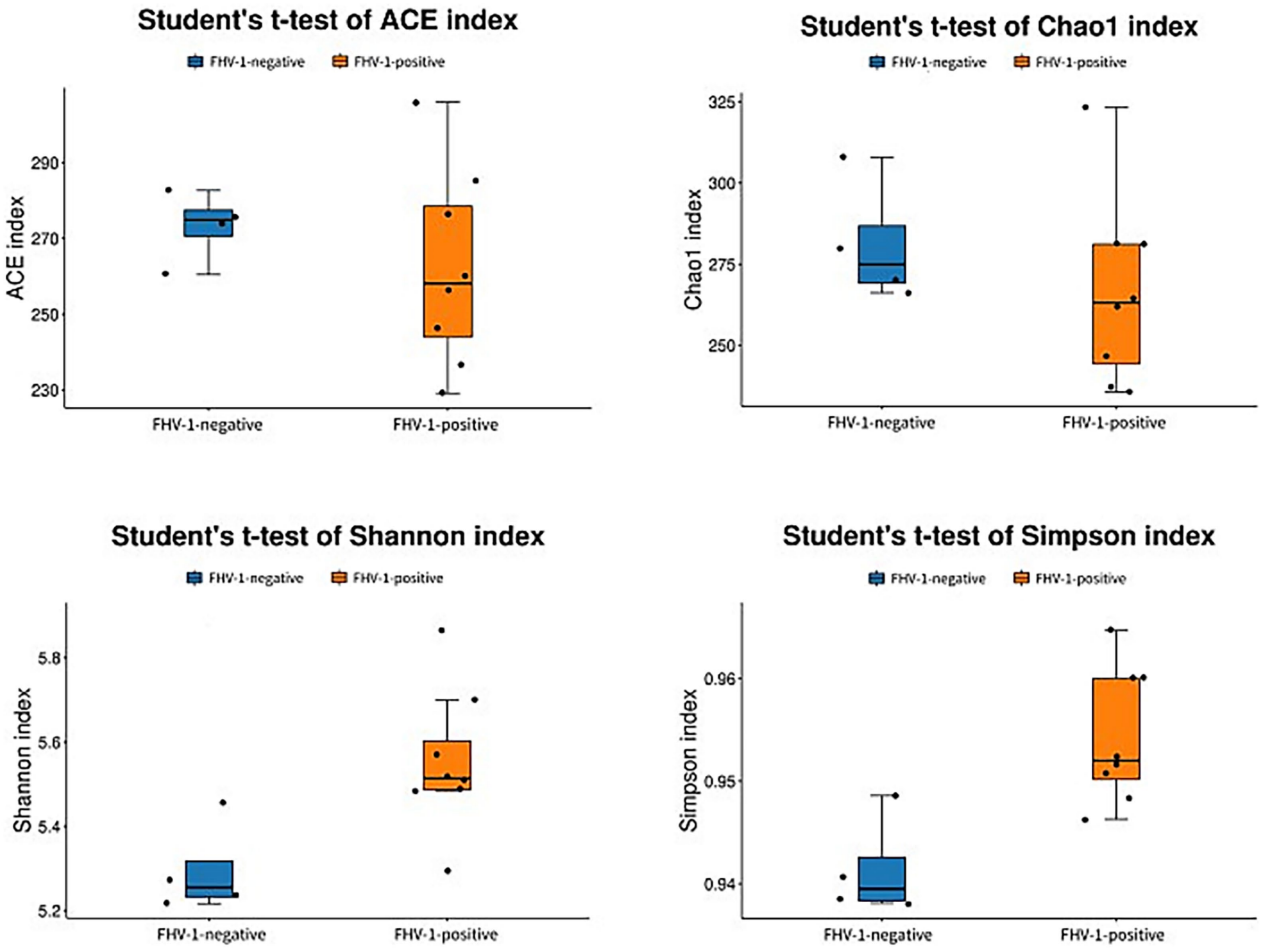
Analyses diagram of alpha diversity index.

Alpha diversity analysis demonstrated significant microbial community differences between groups (Figure 2). FHV-1-positive group exhibited significantly higher Shannon (*p* < 0.05) and Simpson indices (*p* < 0.01) compared to FHV-1-negative group, indicating enhanced microbial diversity in infected individuals. However, no significant intergroup differences were observed in Chao1 (*p* > 0.05) or ACE indices (*p* > 0.05), suggesting comparable species richness between groups (Table 3).

**Table 3:** Analysis of differences in alpha diversity indices.

Index	FHV-1-positive group (*n* = 8, mean ± SD)	FHV-1-negative group (*n* = 4, mean ± SD)	*t*	*p*-value
Feature	242.25 ± 18.05	243.00 ± 7.35	−0.078	0.939
ACE	261.99 ± 25.87	273.26 ± 9.30	−0.828	0.427
Chao1	266.46 ± 29.05	281.03 ± 18.86	−0.901	0.389
Simpson	0.95 ± 0.01	0.94 ± 0.00	3.453	0.006**
Shannon	5.55 ± 0.17	5.30 ± 0.11	2.755	0.020*
PD_whole_tree	21.81 ± 3.69	23.34 ± 3.45	−0.688	0.507
Coverage	1.00 ± 0.00	1.00 ± 0.00	1.279	0.23

*p* > 0.05 indicates no significant difference; *p* < 0.05 indicates a significant difference. *p < 0.05; **p < 0.01.

### 3.3 Beta diversity

**Figure 3:**
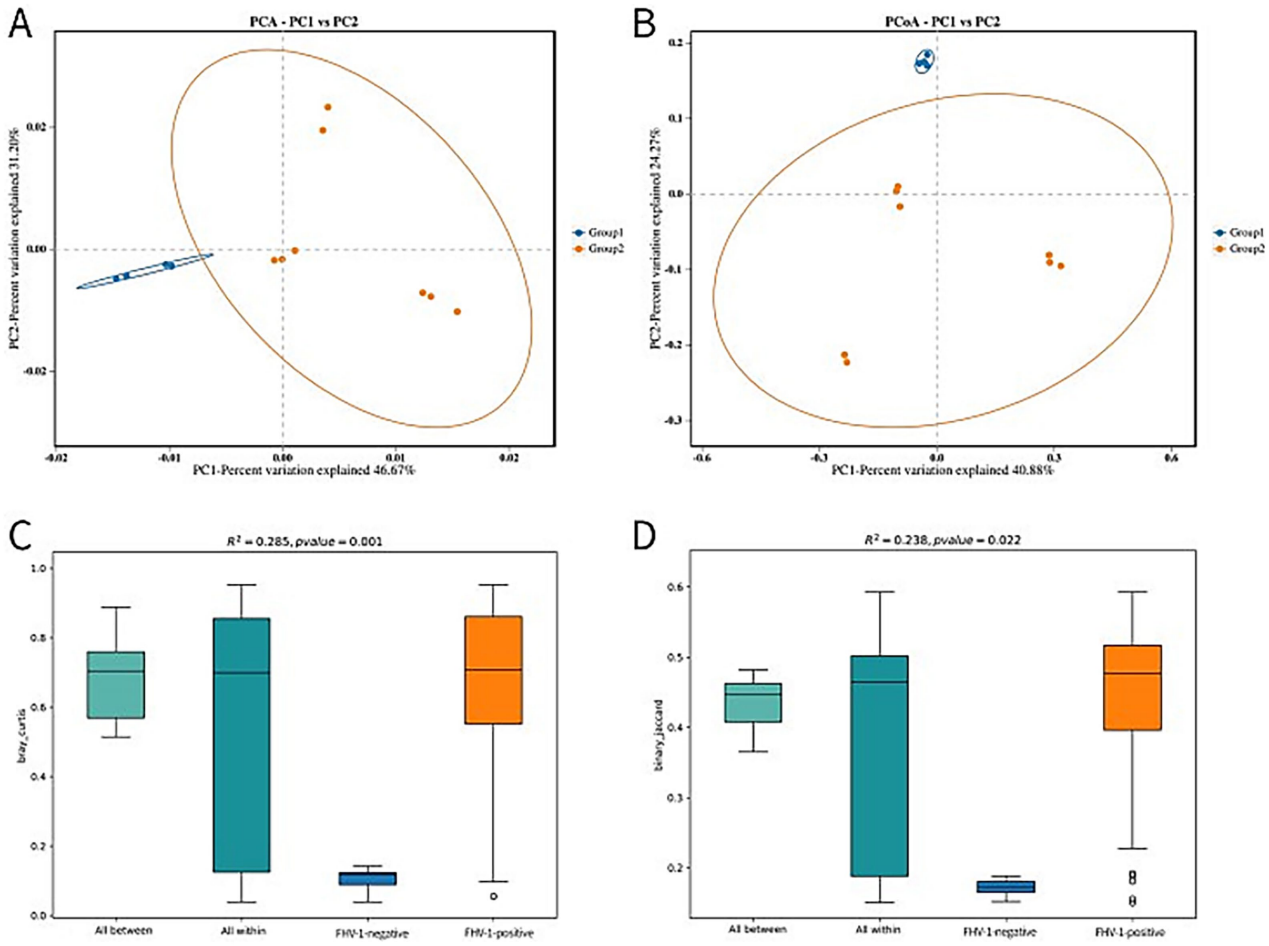
Beta diversity analysis plots. **(A)** PCA analysis plot. The first two principal components account for 46.67% (PC1) and 31.20% (PC2) of the total variation. **(B)** PCoA analysis plot. The first two principal components account for 40.88% (PC1) and 24.27% (PC2) of the total variation. **(C)** PERMANOVA analysis box plot (based on Bray-Curtis distance). **(D)** PERMANOVA analysis box plot (based on binary Jaccard distance).

Principal component analysis (PCA) and Principal coordinates analysis (PCoA) revealed distinct microbial community structures between groups (Figures 3A,B). The FHV-1-negative group showed tight clustering with minimal intra-group variation, while the FHV-1-positive group exhibited significantly greater dispersion, indicating substantial compositional dissimilarities.

The PERMANOVA analysis results demonstrated differential sensitivity of distance metrics in detecting microbial community variation. Analysis using Bray–Curtis dissimilarity showed highly significant differences (*R*^2^ = 0.285, *p* = 0.001) (Figure 3C), whereas the binary Jaccard metric yielded more modest but still statistically significant separation (*R*^2^ = 0.238, *p* = 0.022) (Figure 3D). A larger *R*^2^ indicates a higher degree of explanatory power of group partitioning for the observed differences, reflecting greater divergence between groups, while a *p*-value < 0.05 signifies high reliability of the test results.

### 3.4 Taxonomic composition and differential abundance

**Figure 4:**
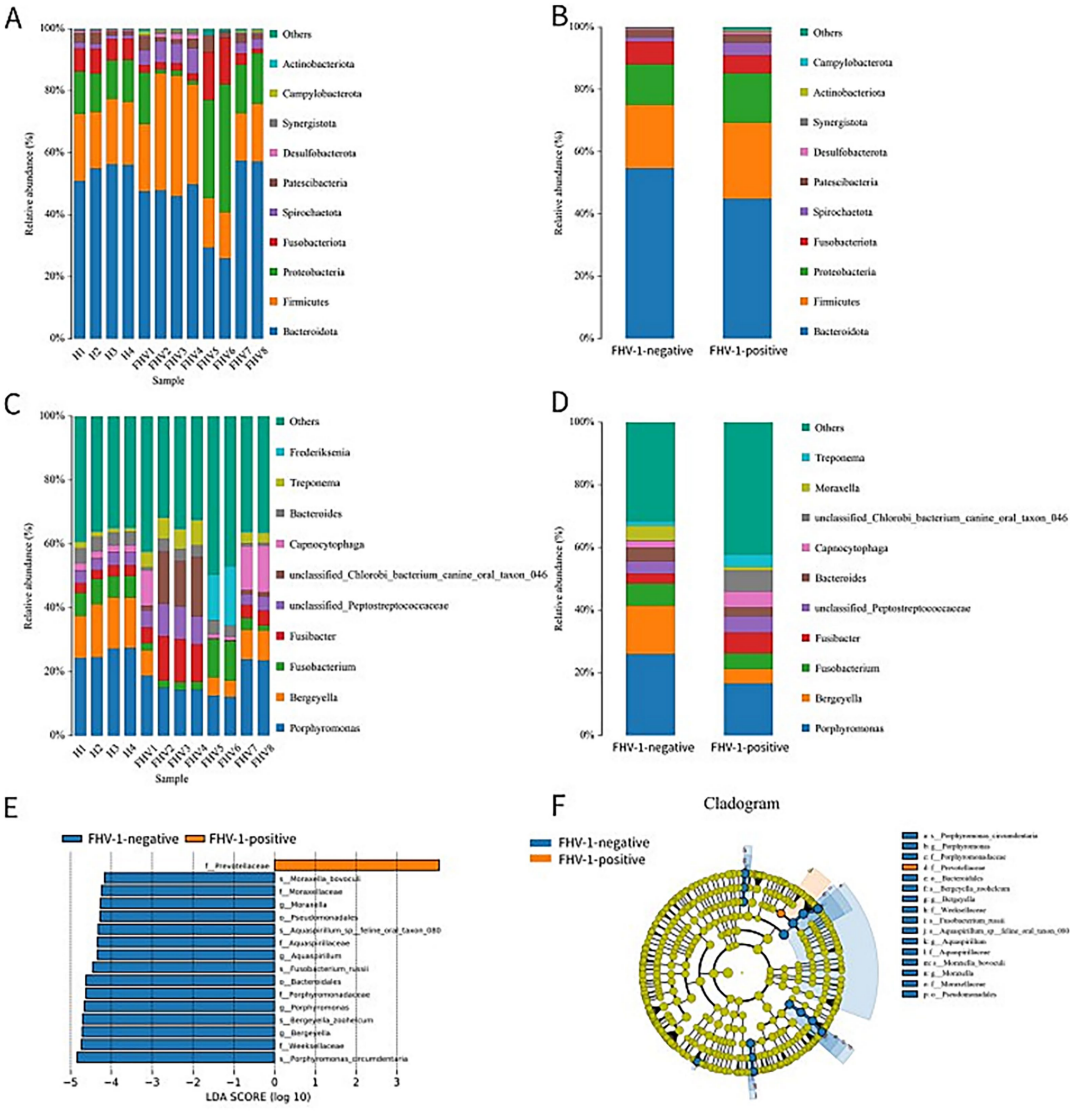
Species classification composition diagrams. **(A)** Species distribution diagram of each sample at the phylum level. **(B)** Inter-group species distribution diagram of FHV-1-positive and FHV-1-negative at the phylum level. **(C)** Species distribution diagram of each sample at the genus level. **(D)** Inter-group species distribution diagram of FHV-1-positive and FHV-1-negative at the genus level. **(E)** LDA scores of significant taxa (LDA > 4, *p* < 0.05). **(F)** Cladogram showing phylogenetic distribution of differentially abundant taxa (colored by enriched group; yellow = non-significant).

The top 10 phyla in terms of species abundance are *Bacteroidota*, *Firmicutes*, *Proteobacteria*, *Fusobacteriota*, *Spirochaetota*, *Patescibacteria*, *Desulfobacterota*, *Synergistota*, *Campylobacterota*, and *Actinobacteriota* (Figures 4A,B). Among them, the abundance of *Actinobacteriota* in the FHV-1-positive group is significantly higher than that in the FHV-1-negative group (0.34% ± 0.05% vs. 0.17% ± 0.03%, *p* = 0.0433) (Table 4).

**Table 4:** Analysis of relative abundance of microbiota at phylum level between two groups of samples.

Phylum	FHV-1-negative (mean ± SE)	FHV-1-positive (mean ± SE)	*p*-value
*Bacteroidota*	54.52% ± 1.24%	45.11% ± 4.11%	0.1487
*Firmicutes*	20.34% ± 0.76%	24.44% ± 3.63%	0.4566
*Proteobacteria*	12.98% ± 0.33%	15.68% ± 5.22%	0.7290
*Fusobacteriota*	7.35% ± 0.27%	5.66% ± 2.11%	0.5925
*Spirochaetota*	1.44% ± 0.19%	4.04% ± 1.06%	0.1221
*Patescibacteria*	2.34% ± 0.63%	2.79% ± 0.53%	0.6195
*Desulfobacterota*	0.47% ± 0.05%	0.58% ± 0.23%	0.7562
*Synergistota*	0.21% ± 0.01%	0.51% ± 0.16%	0.2098
*Campylobacterota*	0.10% ± 0.02%	0.40% ± 0.13%	0.1509
*Actinobacteriota*	0.17 ± 0.03%	0.34% ± 0.05%	0.0433*

*p* > 0.05 indicates no significant difference; *p* < 0.05 indicates a significant difference. *p < 0.05.

The top 10 genera in terms of species abundance are Porphyromonas, Bergeyella, Fusobacterium, Fusibacter, unclassified_Peptostreptococcaceae, unclassified_Chlorobi_bacterium_canine_oral_taxon_046, Capnocytophaga, Bacteroides, Treponema, and Frederiksenia (Figures 4C,D). At the genus level, *Porphyromonas* (16.77% ± 1.66% vs. 25.88% ± 0.86%, *p* = 0.0043), *Bergeyella* (4.68% ± 1.45% vs. 15.38% ± 0.76%, *p* = 0.0006) showed marked reductions in the FHV-1-positive group (Table 5). These reductions may disrupt commensal microbiota-mediated immune surveillance, potentially facilitating secondary infections.

**Table 5:** Analysis of relative abundance of microbiota at genus level between two groups of samples.

Genus	FHV-1-negative (mean ± SE)	FHV-1-positive (mean ± SE)	*p*-value
*Porphyromonas*	25.88% ± 0.86%	16.77% ± 1.66%	0.0043**
*Bergeyella*	15.38% ± 0.76%	4.68% ± 1.45%	0.0006**
*Fusobacterium*	7.11% ± 0.26%	4.86% ± 1.61%	0.3564
*Fusibacter*	3.16% ± 0.14%	6.69% ± 2.00%	0.2499
*unclassified_Peptostreptococcaceae*	3.86% ± 0.12%	5.25% ± 1.43%	0.5188
*unclassified_Chlorobi_bacterium_canine_oral_taxon_046*	0.15% ± 0.01%	6.70% ± 2.88%	0.1477
*Capnocytophaga*	2.09% ± 0.04%	5.18% ± 2.32%	0.3793
*Bacteroides*	4.45% ± 0.14%	2.81% ± 0.52%	0.0552
*Treponema*	1.43% ± 0.19%	3.94% ± 1.03%	0.1236
*Frederiksenia*	0.43% ± 0.03%	4.36% ± 2.65%	0.3315

*p* > 0.05 indicates no significant difference; *p* < 0.05 indicates a significant difference. **p < 0.01.

LEfSe analysis revealed that the family *Prevotellaceae* (LDA score> 4, *p* < 0.05) was significantly enriched in the FHV-1-positive group compared to FHV-1-negative group (Figure 4E), with its taxonomic hierarchy further illustrated in the cladogram (Figure 4F).

## 4 Discussion

Our study revealed a statistically significant elevation in microbial alpha diversity in FHV-1-positive cats, as demonstrated by increased Shannon (*p* = 0.02) and Simpson (*p* = 0.006) indices compared to their FHV-1-negative counterparts. This observed shift in microbial diversity likely results from the complex interplay between virus, host, and microbiota dynamics (13). Specifically, FHV-1-induced mucosal damage may disrupt colonization resistance mechanisms, thereby facilitating opportunistic pathogen invasion and proliferation. Furthermore, the virus-mediated immunosuppression appears to compromise host microbial regulation, permitting expansion of typically commensal or low-abundance bacterial species. We hypothesize that the increased alpha diversity in FHV-1-positive cats may be related to the risk of secondary infection. Previous studies have shown that viral infections can disrupt mucosal barriers and increase microbial diversity, thereby promoting secondary bacterial colonization (14). To minimize sampling bias, all samples were collected from the same anatomical site using sterile swabs, and samples were only included if collected within 24 h of clinical symptom onset to reduce the impact of disease progression on microbial composition.

The beta diversity analysis clearly showed a significant alteration in the microbial community structure in FHV-1-positive group. The PCA, PCoA and PERMANOVA analysis results indicated that the microbiota of the FHV-1-positive group was more dispersed and distinct from that of the FHV-1-negative group. This structural change suggests that FHV-1 infection remodels the upper respiratory tract microbiota, potentially affecting its normal functions.

The significant increase in the abundance of *Actinobacteriota* at the phylum level in the FHV-1-positive group is particularly noteworthy. *Actinobacteriota* are known to produce a variety of bioactive compounds, including antibiotics and immunomodulatory substances (15). In the context of FHV-1 infection, the increased abundance of *Actinobacteriota* might represent an adaptive response of the microbiota to combat the viral infection (16). Conversely, the significant reduction in the abundance of *Porphyromonas* and *Bergeyella* at the genus level in the FHV-1-positive is also of great interest. The altered microenvironment caused by FHV-1 infection, such as changes in pH, oxygen concentration, or the availability of nutrients, may no longer be favorable for their growth and survival (17). However, the reduction in these genera that are potentially pathogenic does not necessarily guarantee a beneficial outcome. It could disrupt the normal ecological balance of the microbiota, leading to the overgrowth of other opportunistic pathogens (18, 19).

The identification of *Prevotellaceae* as a biomarker for the FHV-1-positive group by LEfSe analysis provides a new perspective on the role of specific microbial families in FHV-1 infection. *Prevotellaceae* have been associated with various physiological and pathological processes in the respiratory tract (20, 21). In this study, its increased abundance in the infected group suggests that it may play a role in the response to FHV-1 infection. It could be involved in modulating the host immune response, competing with other pathogens for resources, or adapting to the altered microenvironment caused by the virus (22, 23).

FHV-1 infection drives microbial community changes through a cascade of interconnected biological events. The virus initially damages mucosal epithelial cells through cytolytic replication, compromising both physical barriers and chemical defenses like mucins and antimicrobial peptides (24). Simultaneously, FHV-1 orchestrates a complex immune modulation, first suppressing pro-inflammatory cytokines to evade detection before triggering excessive inflammation. This immunological manipulation disrupts the host’s ability to regulate microbial populations through multiple pathways—impairing phagocytic clearance, altering nutrient competition dynamics, and modifying the availability of critical resources like iron (25). The virus further influences bacterial communities by depleting host cellular resources and modifying surface receptor expression, thereby reshaping the metabolic landscape and adhesion sites within the mucosal environment. These multifaceted interactions between viral pathogenesis, host responses, and microbial ecology collectively explain the observed shifts in both diversity and composition of the respiratory microbiota (26).

## 5 Conclusion

This study provided the first evidence that FHV-1 infection significantly alters the diversity and composition of the upper respiratory tract microbiota in cats. These microbiota changes were likely to play an important role in the pathogenesis of FURTD and offer new targets for the development of microbiota-based therapeutic strategies.

## Data Availability

The original contributions presented in the study are included in the article/Supplementary material, further inquiries can be directed to the corresponding author.

## Footnotes

^1^ http://www.biocloud.net/
